# A qualitative study exploring midlife women’s stages of change from domestic violence towards freedom

**DOI:** 10.1186/s12905-016-0291-9

**Published:** 2016-03-08

**Authors:** June Keeling, Debbie Smith, Colleen Fisher

**Affiliations:** Faculty of Health and Social Care, University of Chester, Bache Hall, Liverpool Road, CH2 1JR Chester, UK; University of Central Lancashire, Preston, UK; School of Population Health, The University of Western Australia, Perth, Australia

**Keywords:** Stages of change, Leaving, Domestic violence, Journey, Escape

## Abstract

**Background:**

Domestic Violence (DV) remains a significant global health problem for women in contemporary society. Existing literature on midlife women’s experiences of domestic violence is limited and focuses on health implications. Leaving a violent relationship is a dynamic process that often requires multiple attempts and separations prior to final termination. The aim of this study was to explore the process of leaving a violent relationship for midlife women.

**Methods:**

This qualitative study involved fifteen women aged between 40–55 who had accessed residential and non-residential community support services for domestic violence within the UK. Community-based support agencies provided these women with access to letters of invitation and participant information sheet explaining the study. The women notified agency staff who contacted the research team to arrange a mutually convenient time to meet within a safe place for both the women and researchers. It was stressed to all potential participants that no identifiable information would be shared with the agency staff. Women were considered survivors of DV if they defined themselves as such. Data were gathered through semi structured interviews, transcribed verbatim and thematically analysed.

**Results:**

Midlife women appear to differ from younger women by transitioning quickly though the stages of change, moving rapidly through the breaking free onto the maintenance stage. This rapid transition is the resultant effect of living with long-term violence causing a shift in the women’s perception towards the violent partner, with an associated reclamation of power from within the violent relationship. A realisation that rapid departure from the violence may be critical in terms of personal safety, and the realisation that there was something ‘wrong’ within the relationship, a ‘day of dawning’ that had not been apparent previously appears to positively affect the trajectory of leaving.

**Conclusions:**

Midlife women appeared to navigate through the stages of change in a rapid linear process, forging ahead and exiting the relationship with certainty and without considering options. Whilst these findings appear to differ from younger women’s process of leaving, further research is needed to explore and understand the optimum time for intervention and support to maximise midlife women’s opportunities to escape an abusive partner, before being reflected appropriately in policy and practice.

**Electronic supplementary material:**

The online version of this article (doi:10.1186/s12905-016-0291-9) contains supplementary material, which is available to authorized users.

## Background

Domestic Violence (DV) remains a significant global health problem for women in contemporary society with almost 30 % of women experiencing physical and/or sexual violence [1]. Domestic violence permeates socio-economic boundaries and communities [2], with a lifetime prevalence rate of 29 % of UK women between 2009–10 [3]. Whilst definitions of DV vary according to country, the UK’s definition includes physical, sexual, psychological abuse, coercive and also controlling behaviours [4].

Existing literature appears to focus largely on younger women’s (under 35 years) reporting of domestic violence when accessing hospital clinics [5–8], and also older women (aged 55 years and over) resident in transition houses [9–11]. Whilst there is no unified definition on what constitutes ‘midlife’, and the differential range between the ‘midlife’ and ‘older women’ age categories are poorly defined, Australian literature identified from a community based and longitudinal population study respectively, as either 45–55 years [12], and 47–52 [13]. The USA literature offers a range of 50 to 64 for women who were recruited at urban emergency department and primary care clinics [14] and 45–64 [15] for women accessed across a range of settings. Within a medicalized focus, ‘midlife’ has been defined as between the ages of 40–60 [16], and 45–64 [17]. However, the current expansion of emerging literature exploring midlife women’s experiences of domestic violence aged 47 to 52 years [13, 18–20], from the USA [15], largely reports on health implications [21].

For women across the age continuum, the effects of DV have been identified, with the consequences of prolonged exposure remaining for a significant period after the violent relationship has ended [Fisher C, Keeling J, Tsou C, Gausia K: Expreiences of Domestic Violecne in Australian Midlife Women. Submitted]. The severity of DV may also act as a contributing factor affecting a woman’s mental and physical health, as will the attending personal, social and economic resources available for that woman [22]. Whilst shared experiences are apparent across ages [15], discrete variances depending on the age of the woman also appear to exist. For midlife women who have experienced a cumulative effect of DV over a long period of time, sense of self, self-esteem and self-worth may be more severely diminished [23] (Keeling J, Smith D, Fisher C: Leaving a violent relationship: perceived need of midlife women in the UK. Submitted) than for their younger counterparts. For older women, mental health issues such as depression and anxiety, poor family relationships, multiple health problems and alcohol dependence may emerge [24]. The corollary of living within a long term violent relationship provides the milieu from which to then explore challenges in the leaving process.

### Leaving a violent relationship

Whilst DV does not usually involve individual acts of violence [25], but a range of physical and non-physical behaviours along with on-going coercive control [4, 26, 27] escalating over a period of time, exposure may gradually undermine a victim’s confidence and ability to leave the relationship. Women from across the age range, supported by an urban domestic violence shelter who did leave were found to be of significant risk of returning to the violent relationship [28]. Impeding a successful separation, a multitude of factors appear accountable including economic dependency on a partner, unemployment and the presence of children for younger women [29], and from data generated from women across the age range, emotional attachment [30], duration and intensity of the relationship [31] and factors associated with socialization [32].

Outcome measures exploring DV often rely on physical [33, 34] and psychometric scales [35, 36]. Whilst useful for prevalence studies and the relationships between (for example) mental health conditions combined with domestic violence, they focus on the resultant effects of perpetrator behaviours, omitting the cognitive changes associated with a woman taking control of her life. In an attempt to translate the process of leaving a violent relationship into a theoretical framework [37, 38], behaviour based approaches [39] within a stages of change model (SOC) [40] have been used. This has been useful in understanding the decision-making processes involved during the journey to safety. To further explain the psychosocial process of leaving, Wuest & Merritt-Gray [41] identified four distinct stages in the process of reclaiming self for rural survivors of domestic violence: counteracting abuse breaking free, not going back and moving on, comparable to Prochaska & Diclemente [40] stages of change model, but contextualised within an abusive relationship. This earlier model in the context of DV may begin with a contemplation stage, the realisation that a relationship might be unhealthy and acknowledging there is a problem. The next phase, determination, is apparent when the women reach a decision to take an action change. The underpinning psychological transition between these two phases is apparent. Preparation leads to a plan of action to change and move away from the violent relationship, followed by action-the actual process of leaving, maintenance stage occurring between 6 months and 5 years in which women continue to live away from the abuser, and finally the relapse stage in which women return to the abuser [30].

The process of returning to the abuser is a cyclical process in which women progress between stages of the cycle but often relapsing back to a previous stage before moving forward again [30], a dynamic process that requires multiple attempts and separations prior to final termination [42]. For younger women with accompanying children, the complexity of leaving a violent relationship may be further intensified due to safeguarding concerns for young children, often necessitating enforced or voluntary interaction with social services to address safeguarding issues [6, 43]. In a retrospective study of 85 women aged 18–55 years recruited from domestic violence shelters and transition housing in the USA, the affective state of women was implicated in the decision to leave a violent relationship, with low levels of anger towards the perpetrator resulting in the woman being less likely to move beyond a pre-contemplative stage [44]. Furthermore, the final decision to leave and the consequent actions may expose associated dangers due to the perpetrator’s possible retaliation and further violence [45]. Davis [46] in discussing the challenges associated with leaving a violent relationship identified strength, survival, resilience and self-protection as important factors and suggested that a process of healing, growth and renewal was required. Strategies such as group practical activities, educational courses, reading, going out and gardening along with a focus on actively attempting to prepare for separation through disclosure to family and friends, or organisations such as Women’s Aid also positively impact on separation [29].

The impetus for this study was the working knowledge that the experiences of midlife women experiencing domestic violence are somewhat limited, and an observation of their apparent unease accessing services that focus on younger women with children. Additionally, midlife women are positioned by having established a home often with adult children with an investment into a long term relationship. This study therefore attempted to illuminate midlife women’s experiences of their journeys away from living with an abusive partner that may then guide practice and enhance our understanding of midlife women’s needs in the postvictimisation phase of a violent relationship, and support a sustained separation from an abusive partner.

## Methods

A qualitative research design using hermeneutic phenomenology [47] provided the theoretical framework for the research methodology, ensuring the women’s lived experience remained the focus of the study.

### Participant recruitment

The nexus between autonomy and informed consent was the paramount consideration in the recruitment strategy for this study, acknowledging the participants’ previous experiences of coercion and control by an abusive partner. We contacted community-based agencies who provide support following domestic violence in the Midlands area of the UK. They made available letters of invitation and participant information sheet to survivors who met the inclusion criteria for this study. It was stressed to all potential participants that the study was entirely independent of the agencies and that no information was being shared with them. Women interested in participating then notified agency staff who contacted the research team to arrange a mutually convenient time to meet within a safe place for both the women and researchers. This process ensured that participation remained a woman’s autonomous decision. All but one of the women who confirmed participation attended for the interview.

### Participants

The inclusion criteria for participation was for women being aged between 40 and 55, and identifying as having experienced domestic violence. Working within the UK definition for midlife as being 40 years old [48] and 55 [49], this study defined midlife as being aged 40–55 to ensure a fracture between older women and midlife women.

### Ethical considerations

Guidance for research involving violence against women was implicit in the development of this study, and focuses on autonomy and confidentiality [50]. Ethical approval was granted by the Faculty of Health and Social Care Ethics Committee, University of Chester, UK, and the Human Research Ethics Committees, The University of Western Australia in Australia. Particular attention to non-maleficence was considered, ensuring that all women had recourse to continuing emotional and practical support in case of distress during or after the interview. No compensation was offered for participation in this study. All participants gave written informed consent to the use of a pseudonym with their data, and for publication of quotes from the interviews. A COREQ checklist is included as an Additional file 1.

### Data collection

Questions in a phenomenological interview address what has been experienced and the context/s that typically influence or affect lived experiences. An interview schedule provided a broad outline for the interviews incorporating enquiry about the women’s feelings towards leaving a violent relationship and the process of leaving, the researchers being mindful to facilitate an interview whereby the woman’s own meaning of her experience was constructed [47]. Demographic data collected included the age of the woman, the length of time within the violent relationship, and number of children. All interviews were conducted within a refuge and audio-recorded with the woman’s permission during 2010 and 2011. Interviews lasted between 30 and 90 min at the women’s discretion. Ensuring anonymity, each woman chose a pseudonym by which to be known. Following the concept of saturation [51], it was anticipated that 10–15 interviews would fulfil this criteria. In line with other qualitative approaches, it was not the intention to use a representative sample with generalisable findings.

### Data analysis

The members of the research team individually read each transcript several times to obtain familiarity with, and an overall feeling for them. Using thematic analysis [52] formulated meanings were clustered into themes. Strategies utilised in the data analysis phase combined to enhance the rigour of the study by ensuring each researcher initially analysed the data independently. The final stage of the analysis resulted in the research team joining and reviewing their themes together to ensure inter-rater reliability. Discussion of the analysis generated shared themes emerging from midlife women’s journey away from domestic violence. A clear audit trail was maintained by the researchers throughout the analysis.

## Results

Fifteen women who met the inclusion criteria participated in the study. Whilst the majority of the women were mothers, it is not known if any were also grandmothers. None of the woman were in employment at the time of the interview.

The women’s stories described experiences of violence, intimidation, isolation and control, these effects arising from the behaviours of perpetrators of DV [27], referred to by Schneider [53] as the generality of coercive control. Through this exercise of power and coercive control, the male perpetrator frequently negates the woman’s ability to leave the relationship [54]. A resultant effect of living with long-term violence appeared to shift the women’s perception towards the violent partner, with an associated reclamation of power from within the violent relationship. The longevity of the abuse also appeared to impact on the women’s feelings towards the partner in two ways; the realisation that rapid departure from the violence may be critical in terms of their safety, and the realisation that there was something ‘wrong’ within the relationship, a ‘day of dawning’ that had not been apparent previously. These issues are presented as three themes ‘longevity of violence’, ‘transformative feelings’ and, ‘leaving the violent relationship’. Despite being reported separately here, they were interrelated and interwoven within the women’s narratives.

### Longevity of violence

All the women in this study experienced DV lasting a period of years, (for one woman, 30 years), with the violence commencing early in the relationship. Physical beatings were often accompanied with sexual violence and other non-physical forms of DV. These acts of violence were comparable to the experiences of younger women [55]. For one woman, the frequency of the violence increased over time:

At first, like I say it [physical violence] was every 4 or 5 months and probably more than that mentally but I realised… by last 10 years the longest it would go would be 8 weeks without me getting a good battering and then in the last 3 years it was every month, I didn’t go more than 4 weeks, you could feel it coming (Molly)

The physical violence was often accompanied with other forms of DV including coercion and control. For many women, ongoing non-physical violence was considered the most debilitating aspect of the relationship with prolonged and pervasive effects.

Out of 20 years that I was with him, I was only allowed out 6 times on my own and then I’d got the kids with me (Molly)

I had known him for 30 years… I moved in with him and everything and my whole life just changed. I lost all my friends, everything, even to the point where he ended up, he was in total control of my life (Paula)

Over the years I have got quieter and quieter and I’ve got nothing to say for myself anymore (Sam)

The outcome of living with DV over a sustained period of time was clearly visible in the women’s narratives, the physical assaults being accompanied by nonphysical violence. For a number of these midlife women, social isolation extended beyond the intimate relationship, extending to enforced withdrawal from friends and family, prevention from leaving the home even for short periods of time and denial of basic communication with others. As one woman noted ‘*he was in total control of my life*’. The excerpts suggest that the women are at the pre-contemplation stage [40], not yet ready to take action. The importance of understanding the nature of the violence and abuse offers us insights into the women’s survival skills (I *have got quieter and quieter and I’ve got nothing to say for myself anymore)* and that are a prelude to beginning the process of leaving.

### Transformative feelings

From experiences of living with a violent partner, with the longevity of abuse and violence came a transformation in the women’s feelings towards their partner. As women noted:

Things fell apart between us because he was drinking and then he’d get nasty and of course after so long of putting up with that, feelings change towards them, you basically hate them (Beth)

I started defending myself. Once I’d had my daughter… I started thinking that I don’t want my daughter thinking this is it, because I had two boys before and with [daughter] I sort of thought, no I am not putting up with this anymore and so I started slowly sort of going against him (Scarlet)

He said ‘what are you doing’ and I said ‘I am packing this suitcase, I am going’ and he begged and pleaded and everything for me to stay and kept saying that he would change. I said ‘but you haven’t changed’… when it comes to actually leaving, I mean for good, it’s a big step but you’ve got to be sure that is what you want to do and at that point for me there was no turning back… I’d said to myself enough is enough, I’ve got to draw the line and I’ve got to do something for me, not for anyone else, for me, to save myself. So I left (Cassie)

An apparent shift in the women’s perceptions towards their male partners may have represented a pre-emptive stage to the women’s decision to leave the violent relationship. Women who maintain negative partner-blaming attributions demonstrate a stronger intent to leave violent relationships compared to women who self-blame [56, 57]. The excerpts, *I started slowly sort of going against him* and *feelings change towards them, you basically hate them,* are indicative of a psychological, rather than physical, active disengagement, an internalisation of shifting feelings towards the abuser. The transition to counteracting abuse may be an iterative cycle demonstrated in diverse and individual ways [41]. The transition from acknowledging the effects of the longevity of relationship abuse to the shift in feelings towards the abuser is indicative of a contemplation stage, not yet ready to take action, but a recognition of an unhealthy relationship [40].

### Leaving the violent relationship

For many women the physical violence escalated until it reached a point such that the women become fearful of losing their lives, leading to a preemptive realization that leaving the relationship was crucial to their survival. Thus, the women’s decision to leave prevailed over their partners’ controlling behaviors, coercion and threats. These findings were also mirrored in Australian midlife women’s experiences [Fisher C, Keeling J, Tsou C, Gausia K: Navigating complexity and the unknown: The journey to violence free lives for Australian midlife women who have experienced domestic violence. Submitted].

If he was going to hit me over something minor, what was he going to do if I told him I’d given my notice in for the job? Then I started getting very scared… I said to [friend] that I think I need to get out (Sam)

I probably had started rebelling because it got to the point where I’d taken it [violence] and taken it until you can’t take anymore… actually left and it was like … I phoned here [refuge] … I was coming here (Susan).

The women’s’ decisions to leave appear to be a ‘one off’ decision, a final act, rather than what Anderson & Saunders [58] describe as a process of leaving, more akin to the experiences of younger women.

After a period of time of getting beaten black and blue, I moved away to another area got away as far as possible, sent him a letter saying “I’ve had enough of him beating me up, so on and so forth and I just sent the keys back in an envelope to say “I’m not coming back”. That was it… It was the Monday morning I got up and thought right, I am no longer going to put up with him and his ways, his attitude and you know. So, I phoned the refuge… I’d said to myself enough is enough, I’ve got to draw the line and I’ve got to do something for me, not for anyone else, for me, to save myself. So I left (Cassie)

So eventually I realised that I had to get away from him and I’ve got to do it, I’ve got to get right away and some help to help me stay away…, he’d gone off to work and I just walked out of the house with just a few things in my bag and went to my youngest son… (June)

For these women, the decision to leave appeared to be due to one of two triggers; realisation that staying within the relationship would potentially be fatal, or for some women, reaching a decision to leave. This stance opposes much of the literature around younger women leaving violent relationships [6, 38] in which they have prolonged and frequent attempts at leaving [42], often supported by statutory agencies involved in safeguarding accompanying children.

The finality in these women’s’ decisions’ was apparent in their narrative ‘*enough is enough’* and ‘*that was it’*. Both Prochaska & Diclemente [40] and Wuest & Merritt-Gray [41] illuminate the cyclical process of behavioural change, and for these midlife women leaving an abuser, they appear to transition quickly though the stages of change, moving rapidly through the breaking free onto the maintenance stage. The women talk in terms of finality *So I left* and *That was it*.

## Discussion

The midlife women talked of their experiences of living with the longevity of DV, and how this had affected specific aspects of their life through these years. They shared experiences of physical, social and psychological violence that may be captured in Schneider’s [53] generality of control. Whilst some of the women in this study talked about previous violent relationships, others had not had this experience. It is therefore noteworthy that the apparent change in all these women’s feelings towards their partner appeared to emerge in midlife, and was the pivotal difference between leaving a violence relationship for these women at this age, than for younger women or indeed these women when they were younger. Emanating from the longevity of experiences of DV was an apparent transformation in the women’s feelings towards their partner whereby a strong dislike for the partner emerged. This paradigm shift is not evident in literature surrounding younger women’s escapes from a violent relationship. Indeed, this study suggests that these midlife women’s’ negative feelings were not present prior to this time, with previous positive feelings towards the partner being what Kearney [59] describes as an incongruity of violence as a basic process of enduring love. However this transformation in the women’s reactions to their partner, from compliance to tangible aversion may have been the pre-emptive stage of preparing to test the process of leaving [60]. The longevity of the violence experienced by these midlife women whose grown children had left home appeared to precipitate a shift in the women’s feelings towards their partner, with a resultant reclamation of autonomy. Leaving a violent relationship has long been acknowledged as an interactive process [41], one in which women return to the abuser several times before finally breaking free [61]. Similarly midlife women enter this process but appear to navigate through from thinking about leaving (contemplation phase) to breaking free, in a rapid linear process.

The stages of change model as depicted by Reisenhofer & Taft [37] illuminates the junctures through which women move from living with a violent partner to finally reaching the exit stage; precontemplation, contemplation, consideration of options (planning), action (leaving) and embracing strategies to live free from violence. Through interaction with advocacy workers [Fisher C, Keeling J, Tsou C, Gausia K: Navigating complexity and the unknown: The journey to violence free lives for Australian midlife women who have experienced domestic violence sumitted] women may be facilitated to conceptualize their experiences as domestic violence, and then supported through the subsequent stages of planning, leaving and living violence free.

In the absence of support from advocacy workers, the emerging catalyst for the UK midlife women to begin their stages of change appeared to originate from a transformation in their feelings towards their violent partner, in what may be considered as the contemplation stage. However instead of then continuing with the subsequent ‘consideration of options (planning)’ stage, these women forged ahead and left their relationships without considering their options ‘*I just walked out of the house’.*

Kearney [59] suggested that this shift in the perspective of the violent relationship may have resulted in the women redefining their situations as unacceptable. Whilst leaving a violent relationship has been identified as a dynamic process that requires multiple attempts and separations prior to final termination [42], these midlife women’s narratives suggest a dissimilar approach, one of finality. For women in younger and midlife age groups, leaving a relationship appears to be segregated acts. Younger women with children are often supported by social workers, their separation from a violent partner oscillating between being voluntary with unsuccessful attempts to leave or through coercive acts employed by social workers [6]. Despite the impending economic hardship and loss of domestic stability on leaving a long term relationship [Fisher C, Keeling J, Tsou C, Gausia K: Navigating complexity and the unknown: The journey to violence free lives for Australian midlife women who have experienced domestic violence. Submitted], the midlife women forged ahead with their plan to leave with a certain finality, and none spoke of their anticipation of leaving their home or material possessions. Whilst such a reasoned approach [58] to actually leaving the violent relationship may have salience, it does not extend to midlife women’s continued absence from the violent relationship.

## Conclusion

The aim of this study was to explore midlife women’s journeys away from domestic violence. Existing literature highlights that whilst women eventually manage to escape their abusive relationships, for younger women this may take repeated attempts. For these midlife women, their decision to leave, appears to be unprompted by external agencies. Additionally, the women appear to progress rapidly through the stage of change cycle at differing intervals to other women: a long latent pre contemplative phase [37, 40] preceding a rapid navigation through the subsequent phases in a linear process leading to breaking free from the abuser. This opposes the literature following a younger woman’s iterative cycle of leaving with recurrent returns to the violent relationship [61].

Whilst these findings differ from younger women’s process of leaving, further research is needed to explore and understand the optimum time for intervention and support to maximise midlife women’s opportunities to escape an abusive partner.

The process of leaving for midlife women can be a long and difficult journey. Exacerbated by the cumulative effects of living with violence over an extended period of time the women’s health and well-being or survival became a primary objective within their life and their decision to leave the relationship.

Consequently, it is important that the experiences of midlife women are clearly articulated and differentiated from those of both younger and older women and built into support agencies to provide care at an opportune moment and capitalize on the breaking free phase. In policy and practice it is also imperative that their experiences are not conflated with other cohorts of women as their experiences do not mirror those of their younger counterparts or of older women. Essentially, the dynamics of leaving and the associated personal, social and economic costs to women differ at varying life stages. To effectively support midlife women deal with DV it is important that responding professionals understand these particularities.

### Limitations

Although a small sample size of fifteen women poses limitations on the generalizability of the data generated, it is hoped that their stories may provide a utility extending to a wider audience and a milieu for further research in this area. All the women participants at the time of interview had accessed support services. Further research is now required to explore women’s experiences in the absence of such support. Due to limitations on the methodology, this paper does not attempt to identify midlife women’s readiness to leave a partner, the pre-emptive stage, and does not attempt to discuss sustained separation. Whilst these midlife women appear to experience a long pre-contemplation phase, followed by a rapid personal journey through the stages of change cycle leading to safety, the authors suggest further research would be beneficial to explore this more fully.

The main limitation is the narrow context of the study as the sample was drawn from a predominantly white British population who had left a violent heterosexual relationship. We acknowledge that whilst domestic violence extends across all relationships, we were not able to extend the sample diversity, raising questions around accessibility of mainstream DV services to diverse women.

### Availability of data and materials section

The complete transcripts supporting the conclusions of this article are not available due to the potential risk of the identification of individuals.

## Additional file

Additional file 1: Consolidated criteria for reporting qualitative studies (COREQ): 32-item checklist (Tong, Sainsbury & Craig, 2007). (DOCX 15 kb)
