# Supplementary material for: A qualitative study exploring midlife women’s stages of change from domestic violence towards freedom
Source: BMC Womens Health. 2016 Mar 8;16:13. doi: 10.1186/s12905-016-0291-9 (PMC4784319; doi:10.1186/s12905-016-0291-9)
Supplement: Additional file 1: — Consolidated criteria for reporting qualitative studies (COREQ): 32-item checklist (Tong, Sainsbury & Craig, 2007). (DOCX 15 kb) [file 12905_2016_291_MOESM1_ESM.docx]

A qualitative study exploring midlife women’s stages of change from domestic violence towards freedom.

Consolidated criteria for reporting qualitative studies (COREQ): 32-item checklist (Tong, Sainsbury & Craig, 2007)

| No | Item | Description |
| --- | --- | --- |
| **Domain 1: Research team and reflexivity** | | |
| **Personal Characteristics** | | |
| 1 | Interviewer/facilitator | June Keeling, Debbie Smith, Colleen Fisher |
| 2 | Credentials | Included in the manuscript |
| 3 | Occupation | Included in the manuscript |
| 4 | Gender | All female |
| 5 | Experience and training | Included in the manuscript |
| **Relationship with participants** | | |
| 6 | Relationship established | No relationship established prior to study commencement |
| 7 | Participant knowledge of the interviewer | Participants aware through letter of invitation and Participant Information Sheet reasons for doing the research |
| 8 | Interviewer characteristics | None |
| **Domain 2: study design**  **Theoretical framework** | | |
| 9 | Methodological orientation and Theory | Phenomenology chosen methodological orientation underpinning the study, made clear in paper |
| Participant selection | | |
| 10 | Sampling | Participants selected using purposive sampling |
| 11 | Method of approach | Participants approached face-to-face |
| 12 | Sample size | 15 |
| 13 | Non-participation | 1 participant dropped out. Reason unknown. |
| Setting | | |
| 14 | Setting of data collection | Within a refuge |
| 15 | Presence of non-participants | No-one else was present in interviews apart from researcher and participant. |
| 16 | Description of sample | Gender and age |
| Data Collection | | |
| 17 | Interview guide | An interview schedule provided a broad outline for the interviews, pilot tested once |
| 18 | Repeat interviews | No repeat interviews carried out |
| 19 | Audio/visual recording | Research used audio recording to collect the data with participant’s informed consent |
| 20 | Field notes | Brief field notes made during interview |
| 21 | Duration | Interviews lasted between 30 and 90 minutes at the women’s discretion |
| 22 | Data saturation | Data saturation discussed within team |
| 23 | Transcripts returned | No transcripts returned to participants for comment and/or correction. This was not possible given the temporary residency of the participants. |
| **Domain 3: analysis and findings**  **Data analysis** | | |
| 24 | Number of data coders | 3 |
| 25 | Description of the coding tree | Did authors provide a description of the coding tree? |
| 26 | Derivation of themes | Themes derived from the data |
| 27 | Software | Microsoft word used to store transcripts |
| 28 | Participant checking | No-not possible given the transient nature of the women’s access to refuge |
| **Reporting** | | |
| 29 | Quotations presented | Participant quotations presented to illustrate the themes with reference to a pseudonym. |
| 30 | Data and findings consistent | There was consistency between the data presented and the findings |
| 31 | Clarity of major themes | Major themes clearly presented in the findings |
| 32 | Clarity of minor themes | No |
